# Susceptibility of Eggs and Adult Fecundity of the Lesser Grain Borer, *Rhyzopertha dominca*, Exposed to Methoprene

**DOI:** 10.1673/031.008.4801

**Published:** 2008-09-05

**Authors:** Y. Chanbang, F. H. Arthur, G. E. Wilde, J. E. Throne, Bh. Subramanyam

**Affiliations:** ^1^ Chiang Mai University, Chiang Mai, Thailand; ^2^ USDA-ARS Grain Marketing and Production Research Center, 1515 College Avenue Manhattan, KS, 66502; ^3^ Department of Entomology, Kansas State University, 123 W. Waters Hall, Manhattan, KS, 66506-4404; ^4^ Department of Grain Science and Industry, Kansas State University, 210 Shellenberger Hall, Manhattan, KS, 66506-4404

**Keywords:** protectant, neonates, IGR

## Abstract

A series of tests were conducted to determine the susceptibility of eggs and neonates of the lesser grain borer *Rhyzopertha dominica* (F.) (Coleoptera: Bostrichidae = Bostrychidae), exposed to the insect growth regulator, methoprene, on filter paper and on rough rice. In the first test, the hatch rate of eggs exposed on filter paper treated with methoprene at the label rate of 0.003 mg [AI]/cm^2^ when used as a surface treatment in structures was 52.0 ± 7.3% compared to 93.0 ± 3.3% on untreated controls. In the second test, eggs were exposed to a dose-response series of 0.00003 to 0.03 mg[AI]/cm^2^. Egg hatch was directly proportional to concentration and ranged from 85.0 ± 2.0% on untreated controls to 26.7 ± 8.3% at the highest concentration tested. In the third test, 1 ppm of methoprene was sprayed on long grain rough rice (paddy) (Cocodrie variety), and then individual kernels were cracked and an egg of *R. dominica* was placed directly on the kernel. On untreated rice kernels, 67.5 ± 11.6% of the eggs hatched and were able to bore inside, and all of these larvae emerged as adults. In contrast, 40.0 ± 5.3% of the eggs placed on treated cracked kernels were able to develop to where the larvae were visible through X-ray detection, but none emerged as adults. In the final test, newly-emerged adults were exposed on rough rice treated with 1 ppm methoprene. The number of eggs from adults on untreated rice was 52.1 ± 4.3 eggs per female, and on treated rice the average egg production was 12.5 ± 1.1 eggs per female. Methoprene applied on a surface or on rough rice affected development of egg hatch also reduced fecundity of parent adults exposed on the treated rough rice.

## Introduction

*Rhyzopertha dominica* (F.) (Coleoptera: Bostrichidae = Bostrychidae), the lesser grain borer, is an internal feeder of various stored raw grains. Females lay eggs singly or in a row on the exterior of the kernel. Eggs hatch and the active neonate bores inside a kernel, where it completes development to the adult stage (Arbogast 1991). Upon reaching the adult stage, the insect emerges from the kernel and creates a large exit hole (Potter 1935; Rees 1995). Therefore, the best method to manage this pest is to control adults before they colonize and reproduce in the grain, or control neonates before they enter the kernels. Applying a grain protectant may offer long-term protection against adults and neonates of *R. dominica* in stored grain.

Insect growth regulators affect development of immature insects, including *R. dominica.* Methoprene is an insect growth regulator that is an analog of juvenile hormone used in insect control. It affects the egg and larval stages of stored- product insects and will suppress progeny production of *R. dominica* (Oberlander et al. 1997; Main and Mulla 1982). Reductions of 99% or complete suppression have been reported from various studies in which *R. dominica* have been exposed to treated wheat (Bengston 1987; Samson et al. 1990). Although insect growth regulators do not normally affect adult insects, Amos and Williams (1977) reported low levels of adult mortality in *Sitophilus oryzae* (*L.*), the rice weevil, *S. grananus*, the granary weevil, and *R*. *dominica* exposed on treated grains. Similarly, Arthur (2004) reported mortality of *R*. *dominica* adults exposed to dust and liquid formulations of methoprene.

Rice is an important economic crop throughout much of southeastern Asia, China, Japan, parts of Europe and the United States of America (Moldenhauer et al. 2004). *Rhyzopertha dominica* is a common pest of all stored grains, including rice, and is found throughout most of the regions where rice is grown and stored. Economic losses from infestation of *R. dominica* include actual damage, weight loss of kernels, and product contamination (Howell and Cogburn 2004). Rice is stored as rough rice (paddy) before being de-hulled and milled for direct consumption or other end use products. Rough rice kernels have characteristics different from other grains, and consist of the palea and lemma with silica or silicic acid on the outer part of the hull (Champagne et al. 2004). The texture of the hull is rough, which could affect the adherence of residual insecticides, such as methoprene, used to control *R. dominica* and other stored-grain insects.

Methoprene has been used as a grain protectant for many years in Australia as part of pest management plans to control *R dominica* in stored wheat (Daglish et al. 1995; Daglish 1998). In the United States, mtehoprene was re-introduced into the stored-product market in 2002 by Wellmark International (www.wellmarkinternational.com) and marketed the commercial formulation Diacon II®. A series of experiments was conducted to determine: 1) the effect of methoprene on hatch rate when eggs of *R. dominica* were exposed on filter paper or directly on rough rice treated with methoprene; and 2) the effect of methoprene on *R. dominica* after exposure to rough rice treated with methoprene.

## Materials and Methods

An initial test was conducted to determine susceptibility of eggs of *R. dominica* to methoprene (commercial formulation Diacon II, s-isomer of methoprene, isopropyl (2E, 4E, 7S)-11-methoxy-3,7,11-trimethyl-2-4-dodecadienoate, 33.6% active ingredient (AI). All adults used in our studies were obtained from colonies reared on Cocodrie var. long-grain rice. This test was conducted at 32°C to ensure a quick hatch of eggs. The relative humidity was controlled using a saturated sodium chloride solution to maintain humidity at an approximate range of 65–70% (Greenspan 1977), hereafter the percentage RH will be cited as 68%. The RH was maintained using plastic box chambers measuring 26 × 36.5 × 15 cm, with plastic waffle-type grids at the bottom, and described in detail by Arthur and Throne (2003) and Arthur (2004). Methoprene solutions (Diacon II^®^ , 33.3% EC, 300mg/ml active ingredient [AI]) were sprayed onto filter paper (12.5 cm diameter) in proportion to the label rate of 1 ml of product in 3.8 liters to cover 94 m^2^, or 0.003 mg [AT]/cm^2^. Liquid was sprayed on the filter paper (0.5 ml) using a Badger 100 Artist airbrush (Badger Airbrush, www.badgerairbrush.com) to mist the solution directly onto the filter paper. The nozzle projected the spray directly onto the filter paper from a height of about 25 cm. Untreated papers were sprayed with 0.5 ml of distilled water.

About 500 unsexed two-week-old adults were obtained from the laboratory colonies, put in a 0.95 L jar with about 200 g of rough rice, and held for two days at 28°C and 68% RH Rice kernels and adults were sieved with # 12 and 30 sieves (openings 1.70 and 0.59 mm, respectively), and eggs and frass were collected in a solid pan underneath the sieves. Egg collection was done using a modification of a method used by Elek (1994). The sieved material was placed in a 13 cm diameter Petri dish, the dish was placed under a stereo microscope (Leica MZ6, Leica Microsystems, www.leica-microsystems.com), and individual eggs were collected using a camel hair brush. The brush was dipped in clean tap water and dried with a paper towel to reduce static electricity. The dish was then placed under a second stereo microscope (Wild M-8, www.leica-microsystems.com) to ensure that the egg had been placed on the treated paper.

Ten 2-day-old eggs were placed on the treated filter paper, which was placed in a 13-cm-diameter Petri dish. Ten replicates were conducted on the same day in a completely randomized design. The insects were randomly picked from different colonies for each replicate, and methoprene was also prepared individually for each replicate. The number of eggs hatched was counted 7 days after exposure on the papers, and the data were analyzed using the General Linear Models Procedure (SAS Institute 2001, www.sas.com) with number of hatched larvae as the response variable. A significance level of *P* < 0.05 was used for comparisons.

A dose-response test was conducted with concentrations of 0 (untreated control), 0.00003, 0.0003, 0.003, and 0.03 mg [AI]/cm^2^ methoprene applied to filter paper as described above. The solutions were prepared and the paper was treated as described above. Treated papers were air-dried for 1 h and afterwards a 6.5- mm-diameter circle was cut out of the paper. One of these treated circles were placed in 20 wells of a 96-well plate. A single 2-day-old *R. dominica* egg was placed in each well, along with crumbed rice (brown rice ground through a coffee grinder) as a food source for the neonates. All 96-well plates were put in a box of sodium chloride solution to maintain RH. at 68% as described above, and the box was incubated at 32°C. Each replicate consisted of 20 wells, with five replicates on successive days. Neonates on the filter paper were counted after 7 days. The test was analyzed using the Probit procedure (SAS Institute 2001) with the number of unhatched eggs (number of total eggs minus number of hatched larvae) as the response variable to determine mean effective dose (ED_50_).

To assess the effectiveness of methoprene on eggs of *R. dominica* when applied directly to rough rice, solutions were formulated as described above to give 1 ppm when sprayed at the rate of 0.7ml/kg on 500 g of Cocodrie variety long-grain rough rice. The rice was laid out in a thin layer on a 0.6 by 0.3 m surface, and solutions applied using the airbrush described in the first two experiments. Untreated controls were sprayed with tap water at the same rate. This thin layer of treated kernels was about 60 mm in height so that most of the kernels received the spray application. In addition, after spraying, the kernels were put in a 0.95 L jar and tumbled by hand for about 30 seconds to ensure a thorough mixing. Single kernels were cracked using the coffee grinder and put in 30 of the wells in a 96-well plate. Before placing the kernels in the well, steel wool was used to scratch the sides of the well to reduce static electricity. A 2-day-old egg was then placed on the treated rice kernel in each well. The RH of 68% was controlled using the saturated NaCl solutions as described above, and the well plates were put in individual plastic boxes. In this test, the temperature used was 27°C to conform to the standards used for the rice cultures in our colony incubators.

The experiment was repeated each of the following three weeks for a total of four replicates. Five weeks after placing the eggs on the kernels, the presence of the larval stage inside the kernels was assessed using an X-ray (Throne 1994; Arthur and Throne 2003). All kernels were in a single layer on a cellulose sheet and then placed on the film and exposed for 3 min to an X-ray source (Model 48355A, Faxitron, Hewlett Packard, McMinnville, OR, USA). Infested rice was returned to the well plates after the x-ray pictures were taken and held for an additional three weeks to collect emerged F1 adults. The test was analyzed using the GLM Procedure of SAS, with number of larvae inside kernels and adult emergence as the response variables. A significance level of *P* < 0.05 was used for comparisons.

A final test evaluated effects of adult exposure to methoprene on fecundity. Approximately five hundred 1-2-week-old unsexed adult *R. dominica* were placed in a 0.95 L jar with 300 g of Cocodrie variety long grain rough rice and allowed to oviposit for 5 days. These adults were removed using a #30 sieve (openings 0.59 mm), and the eggs, frass, and fine material were put back into the jar with the rice. Four colony jars were replicated from different colonies for each of four replicates. Jars were held at 27°C and 68% RH for 7 weeks, at which time the insects were in the pupal stage and almost ready to emerge as adults.

Two hundred grams of this infested rough rice from each colony jar was sprayed directly with 1 ppm of methoprene at a rate of 0.4 ml per 200 g of rough rice using the airbrush system described in the previous experiments. The methoprene diluents were prepared separately for each replicate. The treated rice was put back into the jar and hand-tumbled for 30 seconds. Distilled water was sprayed on infested rough rice at the same rate for the untreated controls, and each treatment was replicated four times on successive days. In the next two days, new adults began emerging from the rice kernals. Because the eggs were set up at a specific time, the adult emergence was generally completed during a 3–5 day period. Those adults were allowed to live inside this jar for 2 weeks (after this 3–5 day period of emergence) and then were sieved out of the grain. Ten adults were selected randomly and put in a 29-ml vial with rice which had been treated with methoprene at the same rate and in the same manner as previously described (0.4 ml per 200 g of rough rice). In untreated controls, 10 adults from the untreated colony jar were placed in 29-ml vials with untreated grain. All vials were maintained at 27°C and 68% RH as described for the previous experiment.

In each vial, eggs were sieved out using the #30 sieve described above and counted on 14, 19, and 24 days after emergence; the eggs were not returned to the vial. The original parent adults were sexed to determine the number of eggs or neonates produced per female during the 24 days, using characters of genitalia in males and females (Potter 1935). The data were analyzed as a repeated measure using the Mixed Procedure (SAS Institute, 2001). The Repeated statement was used with Compound symmetry (CS), Autoregressive 1 and Combination of CS and Autoregressive 1 to assess if the time measurement or order of times affected egg collections at 14, 19, and 24 days.

## Results and Discussion

The hatch rate of *R. dominica* eggs of controls was about 80%, which is similar to previous studies (Howe 1950; Elek 1994). All neonates that emerged from eggs in the untreated controls and in the treatments were alive when examined. The hatch rate of eggs exposed on filter paper treated with methoprene at the rate of 0.003 mg [AI]/cm^2^ was 52.0 ± 7.8% compared to 93.0 ± 3.3% on untreated filter paper (*F* = 49.0; d.f. = 1, 9; *P* < 0.01). In the dose-response study, the hatch rate of *R. dominica* eggs on untreated filter paper was 85.0 ± 2.0%, while the hatch rate of eggs exposed on the treated paper decreased with increasing concentration of methoprene (Table 1). Although there are no comparable studies in which *R. dominca* eggs were exposed on a surface treated with methoprene, ovicidal effects were also reported when wheat was treated at an application rate of 1 ppm methoprene, with young eggs being more sensitive than older eggs (Main and Mulla 1982; Staal 1975; Walker and Bowers 1970). The average percentage of *R. dominica* neonates that penetrated the untreated rice kernels and developed to the stage where they were visible on x-rays was 67.5 ± 11.6%, and all of these larvae emerged as normal F_1_ adults. In the methoprene treatment, 40.0 ± 5.3% of the eggs developed to the larval stage; however, none of these larvae emerged as adults. The percentage of larvae present in rough rice in the control was not significantly different from the percentage of larvae in the treatment (*F* = 5.7; d.f. = 1, 3; *P*= 0.09), but the percentage of emerged adults in the treated rice was significantly lower than the number in the control (*F* = 35.4; d.f. = 1, 3; *P* < 0.01). In these tests, larvae that survived exposure at the egg stage and were able to bore inside the kernel did not reach the adult stage. The egg and/or larva apparently had absorbed enough of the methoprene to inhibit normal larval development. Larvae were unable to reach the pupal stage, and no insect-damaged kernels were found in the treatments. Smet et al. (1989) showed that larvae of the confused flour beetle, *Tribolium confusum*, exposed to methoprene during the larval stage died during the pupal stage.

**Table 1.**
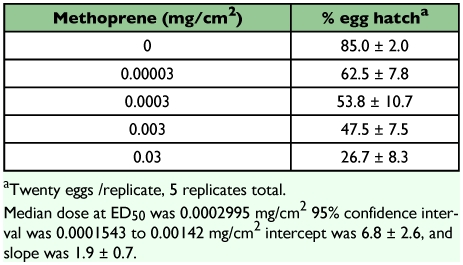
Percentage (mean ± SE) hatch of 2-day-old eggs of R. *dominica* exposed on filter paper treated with methoprene The results were assessed 7 days after eggs were placed on treated filter paper.

There were no time effects with respect to the egg collections on day 14, 19 and 24 (*F*= 0.3; d.f. = 2, 40.1; *P* = 0.71), indicating that the adults produced eggs at a uniform rate during the time periods of 0 to 14, 15 to 19, and 20 to 24 days after emergence. Exposure of adult *R. dominica* to methoprene produced a significant reduction in number of eggs laid on the kernels compared to the untreated controls (*F*= 24.5; d.f. = 1, 21; *P* <0.01, Table 2). The number of eggs laid by treated adults was only 24% of the control. The effect of methoprene on progeny production is also evident from other studies in which adult *R. dominica* have been exposed to wheat treated with methoprene (Arthur 2004; McGregor and Kramer, 1975). Loschiavo (1975) reported reductions in oviposition of *T. castaneum and T. confusum*, while Daglish and Pulvirenti (1997) cite reduced oviposition of *R. dominica* exposed for one week on wheat treated with 0.1 to 4 ppm methoprene. In their test, methoprene applications of 0.01 and 0.25 ppm on wheat reduced progeny by 38.3 to 89.3%, respectively. Our results show similar effects on fecundity of parent *R. dominica* exposed on rough rice treated with methoprene.

**Table 2.**
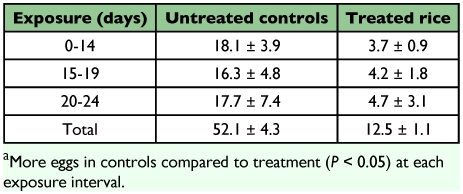
Eggs plus neonates produced per female adult *R*. *dominica* after 14, 19, and 24 days on untreated rough rice or rice treated with 1 ppm methoprene^*a*^.
